# Budd-Chiari syndrome and its associated hepatocellular carcinoma: Clinical risk factors and potential immunotherapeutic benefit analysis

**DOI:** 10.3389/fonc.2022.1075685

**Published:** 2022-12-08

**Authors:** Kang-Shuai Li, Sen Guo, Yu-Xin Chen, Zong-Li Zhang

**Affiliations:** Department of General Surgery, Qilu Hospital, Cheeloo College of Medicine, Shandong University, Jinan, China

**Keywords:** Hepatocellular carcinoma, Budd-Chiari syndrome, malignancy, immunotherapeutic effect, IVC block

## Abstract

**Background:**

Hepatocellular carcinoma (HCC) is a well-described complication of Budd-Chiari syndrome (BCS). However, the risk factors of BCS in developing HCC and clinical characteristics and imaging features of BCS-associated HCC is still to be determined.

**Methods:**

Data from 113 consecutive patients with primary BCS in Qilu hospital were retrospectively studied. The clinical features of 12 HCC patients associated with BCS were also analyzed. Chi-square analysis was performed to analyze the differences in clinical characteristics. The treatment regime and CT imaging features of BCS-associated HCC were also illustrated.

**Results:**

113 consecutive patients admitted to our hospital between January 2009 and June 2016 with a primary diagnosis of BCS were enrolled. 10.6% (12/113) was diagnosed with HCC. The BCS patients were mainly male gender with an average age of 49.2 years. Symptom duration longer than one year exhibited decreased serum ALT and AST and increased ascites ratio. BCS-associated HCC patients were presented with IVC block and stricture of the hepatic venous outflow tract. Patients with HCC were older and showed elevated serum AST and total bilirubin. Most nodules of HCC located in the right posterior lobe with heterogeneous enhancement during the arterial phase and washout during the delayed phase.

**Conclusions:**

The results indicate that BCS patients with IVC block and stricture of hepatic venous outflow tract seem to be associated with HCC. BCS associated HCC nodules exhibited irregular and heterogeneous enhancement in the arterial phase and washout on the delayed phase.

## Introduction

The Budd-Chiari syndrome (BCS) is a group of disorders characterized by hepatic venous outflow obstruction. (1–4) Obstruction of the hepatic venous outflow tract has been reported to be associated with hepatocellular carcinoma (HCC), the most common form of primary liver malignancy. (5–7) However, the incidence rate of HCC in BCS patients varies from country to country, and the predisposing factors associated with the development of HCC are still to be illustrated. (8) Studies have found that following chronic hepatic congestion, the cirrhotic process can develop HCC in patients with BCS, which still needs further evidence. (9)

Ultrasonography, CT, and MRI, in combination with the clinical manifestations, are often applied for the diagnosis of BCS, although angiography is still the gold diagnosis standard. (10) Besides imaging features, the serum α-fetoprotein (AFP) has also been applied to diagnose and screen HCC. There is an increasing acceptance of the diagnosis of HCC using imaging techniques without a biopsy. (11) Although some researchers reported the imaging features of HCC in patients with BCS, typical images on the obstruction site and HCC nodules are still in need. (12) Also, the invasiveness of nodules in BCS-associated HCC patients still needs to be evaluated.

This study retrospectively investigated a cohort of 113 consecutive BCS patients, including 12 BCS-associated HCC patients. This study aimed to analyze the clinical and imaging features of HCC and evaluate the malignancy of nodules in BCS-associated HCC patients. The potential immunotherapeutic benefits were also discussed.

## Materials and methods

We retrospectively studied 113 consecutive patients with a primary diagnosis of BCS who was admitted to our hospital between January 2009 and June 2016. The basic data were acquired by reviewing the patient medical record. The diagnosis of BCS followed the criteria by the European Group for the Study of Vascular Disorders of the Liver. All patients underwent ultrasonography, computed tomography (CT), or angiography to examine the obstruction of hepatic veins, portal veins, or IVC. The diagnosis of BCS was based on imaging showing an obstructed venous outflow tract. The diagnosis of HCC followed the guidelines for diagnosing and treating primary liver cancer in China (2019 Edition) (13). The diagnosis of HCC was based on ultrasonography, CT imaging features, and serum AFP level. Liver nodules were evaluated according to the following imaging features: shape, number, size, location, enhancement on arterial phase, washout on portal venous phase, tumor thrombus of the portal vein, and invasion of the bile duct.

Inclusion criteria of Budd-Chiari syndrome: 1. Previously diagnosed patients; 2. Those admitted to the hospital for treatment with related symptoms of Budd-Chiari syndrome this time; 3. Patients were admitted for other reasons and found-Budd Chiari syndrome during the inspection. Inclusion criteria for Budd-Chiari syndrome with HCC: 1. Those previously diagnosed with Budd-Chiari syndrome, received or did not receive treatment, and were hospitalized for HCC this time; 2. There was no diagnosis of Budd-Chiari syndrome in the past. The patients were admitted for HCC this time and were confirmed to have Budd-Chiari syndrome by systematic examination; 3. The patient was admitted to the hospital for treatment due to symptoms of Budd-Chiari syndrome, and the presence of HCC was confirmed by systematic examination. Exclusion criteria: secondary Budd Chiari syndrome caused by HCC hepatic venous metastasis or inferior vena cava metastasis.

SPSS version 25.0 software was applied for statistical analysis. A Chi-square analysis was performed to analyze the four-fold table data. For cells with theoretical frequency less than 5, Fisher exact test was applied. P<0.05 was considered statistically significant.

## Results

### Clinical features of 113 patients with Budd-Chiari syndrome

The clinical data of patients with Budd-Chiari syndrome in Qilu Hospital of Shandong University was analyzed, and the results were summarized in **Table 1**. The average age of the patients was 49.2 ± 13.4 years. After further dividing the age by the interval of ten years, we found that Budd Chiari syndrome occurred in 0-90 years. Among them, the peak of the incidents occurred between 40 and 50 years old, accounting for about 30% of all patients (Figure not shown). Male patients accounted for 65% of the total number of patients. The proportion of new cases with a symptom duration of less than one year and cases with a symptom duration longer than one year is about 1:1. Among the 113 cases, 5 cases (4.4%) carried the hepatitis B virus, and 1 case (0.9%) had the hepatitis C virus. In terms of hepatic venous outflow tract obstruction, inferior vena cava obstruction and mixed obstruction of inferior vena cava, and hepatic vein obstruction were the main types of obstruction, accounting for 44% and 12%, respectively. There were 9 cases (7.9%) with hepatic encephalopathy and 50 cases (44%) with ascites (**Table 1**).

**Table 1:** Clinical data of 113 cases diagnosed with Budd-Chiari syndrome.

Characteristics	Budd-Chiari syndrome
Age	49.2 ± 13.4
Gender (male: female)	73:40
Symptom duration (<1y: >1y)	55:58
Hepatitis virus-carrying (HBsAg: anti-HCV)	5:1
Hepatic venous outflow tract obstruction subtype	
hepatic vein occlusion	13
obstruction of the inferior vena cava	50
mixed type	50
Hepatic encephalopathy	9
Ascites	50

To determine the effect of the duration time of hepatic venous outflow tract obstruction on liver function, we further compared the liver function of 113 patients with symptoms lasting less than one year and more than one year (**Table 2**). The results revealed no significant differences in age, gender, IVC obstruction, and hepatitis B virus infection between the two groups. The positive rates of ALT, AST, and ascites were significantly different between the two groups. The positive rates of ALT, AST, and ascites in patients with symptoms lasting less than one year were higher than those lasting longer than one year. There were no significant differences in other indicators such as bilirubin, albumin, international standard ratio of prothrombin time (PT-INR), platelet, leukocyte, hemoglobin, and incidence of hepatic encephalopathy. Our results indicate that there is apparent hepatocyte damage in the early stage of hepatic venous outflow tract obstruction, and the incidence of ascites is also high due to portal hypertension. For patients with Budd-Chiari syndrome for more than one year, hepatocyte damage and ascites occurrence are relatively low owing to the potential compensatory mechanism.

**Table 2:** Comparison of primary clinical data between patients with BCS duration time less than one year and BCS duration time more than one year.

	n	BCS duration time		P
		<1year (n=55)	>1year (n=58)
Age		49.5 ± 14.5	48.9 ± 14.5	0.83
Gender				0.08
male	73	31	42	
female	40	24	16	
Hepatic venous outflow tract obstruction subtype				0.60
obstruction of IVC	50	27	23	
hepatic vein occlusion	13	6	7	
Mixed type	50	22	28	
HBsAg				0.67
positive	5	3	2	
negative	108	52	56	
Laboratory examination				
ALT				<0.01
<40 U/L	87	34	53	
>40 U/L	26	21	5	
AST				0.02
<40 U/L	78	32	46	
>40 U/L	35	23	12	
Total bilirubin				0.82
<21 μM/L	44	22	22	
>21 μM/L	69	33	36	
albumin				0.31
>35 g/L	79	36	43	
<35 g/L	34	19	15	
PT-INR				0.99
<1.2	74	36	38	
>1.2	39	19	20	
PLT				0.79
>100*10^12^/L	59	28	31	
<100*10^12^/L	54	27	27	
WBC				0.79
>4*10^9^/L	61	29	32	
<4*10^9^/L	52	26	26	
Hb				0.76
>131 g/L	62	31	31	
<131 g/L	51	24	27	
Hepatic encephalopathy				0.74
+	9	5	4	
–	104	50	54	
ascites				<0.01
+	49	33	17	
–	64	23	41	

The clinical features of 12 HCC patients associated with BCS are shown in **Table 3**. Nine (75%) of the 12 patients were male, and 3 (25%) were female. The average age was 54.4 years (35-70 years). BCS was diagnosed with imaging features (ultrasonography or CT). HCC was diagnosed with imaging features (ultrasonography or CT) and serum AFP level in 10 patients and with histological examinations in 2 patients receiving surgical removal of the nodule. The histological findings of the two samples showed hepatocellular carcinoma and cirrhosis in adjacent parenchyma. The mean duration between diagnosis of BCS and HCC was 17 years (range, 3-40 years). Inferior vena cava block alone was found in 83.3% (10/12) of patients, and hepatic vein and IVC block were observed in 16.7% (2/12) of patients. The serum AFP level was normal in 3 patients with HCC (3.8 ng/ml, 7.9 ng/ml, and 10.3 ng/ml, respectively, normal range < 20 ng/ml), whereas the mean level was 3321 ng/mL (range, 30.1-20643 ng/ml) in the other ten patients. Only one patient was HBV positive, and no patient with HCV positive. Hepatic function reserve was evaluated according to Child-Pugh grades. Seven patients were in A level; 2 patients were in B level, while three were in C level, indicating a poor hepatic function reserve.

**Table 3:** Clinical features of 12 HCC patients associated with BCS.

Pt#	Age	Sex	Duration of symptoms (months)	HBV/HCV	Child-Pugh	AFP(ng/ml)	Site of obstruction
1	35	M	8	-/-	A	249	IVC
2	51	F	NA	-/-	A	–	IVC+HV
3	35	M	NA	-/-	B	7.9	IVC
4	46	M	5	-/-	C	3.8	IVC
5	48	M	40	-/-	A	20643	IVC
6	48	M	19	-/-	A	63.3	IVC
7	43	F	NA	-/-	B	1639	IVC
8	54	M	NA	+/-	A	–	IVC+HV
9	68	F	3	-/-	A	30.1	IVC
10	70	M	34	-/-	C	500	IVC
11	61	M	10	-/-	A	122.5	IVC
12	45	M	NA	-/-	C	10.3	IVC

Pt#, patient number; NA, not available; HBV, hepatitis B virus; HCV, hepatitis C virus; AFP, a-fetal protein; IVC, inferior vena cava; HV, hepatic vein.

To find out the risk factors of liver cancer in patients with Budd-Chiari syndrome, we compared the basic clinical data of 101 patients with Budd-Chiari syndrome and 12 patients with Budd-Chiari syndrome and associated HCC (**Table 4**). The results showed significant differences in age, location of the obstruction, AST, and bilirubin. Still, there were no significant differences in gender, HBsAg, ALT, albumin, PT-INR, platelet, leukocyte, hemoglobin, hepatic encephalopathy, and ascites. The difference in age between the simple group of Budd-Chiari syndrome and the group of Budd-Chiari syndrome associated HCC indicates that liver cancer generally occurs within a few years after the occurrence of Budd-Chiari syndrome. Namely, liver cancer is related to the presence of Budd-Chiari syndrome. Consistent with the literature reports, our study also found that IVC obstruction is associated with the occurrence of liver cancer, but there is no reasonable explanation at present. There was a marginally significant difference in AST and bilirubin (P = 0.05), which might be affected by the nodules of HCC.

**Table 4:** Comparison of clinical data between patients complicated with HCC and not complicated with HCC.

	n	BCS not complicated with HCC (n=101)	BCS complicated with HCC (n=12)	*P*
Age		47.5 ± 13.6	56.2 ± 10.6	0.04
Gender				0.53
male	73	64	9	
female	40	37	3	
Obstruction type				0.01
IVC type	50	40	10	
HV type	13	13	0	
Mixed type	50	48	2	
HBsAg				0.44
positive		4	1	
negative		97	11	
Laboratory examination				
ALT				0.30
<40U/L	88	77	11	
>40U/L	25	24	1	
AST				0.05
<40U/L	78	73	5	
>40U/L	35	28	7	
Total bilirubin				0.05
<21 μM/L	78	73	5	
>21 μM/L	35	28	7	
Albumin				1
>35 g/L	79	70	9	
<35 g/L	34	31	3	
PT-INR				0.75
<1.2	74	67	7	
>1.2	39	34	5	
PLT				0.87
>100*10^12^/L	59	53	6	
<100*10^12^/L	54	48	6	
WBC				0.38
>4*10^9^/L	61	56	5	
<4*10^9^/L	52	45	7	
Hb				1
>131 g/L	62	55	7	
<131 g/L	51	46	5	
Hepatic encephalopathy				1
+	9	8	1	
–	104	93	11	
ascites				0.13
+	50	42	8	
–	63	59	4	

### Imaging features of hepatic venous outflow tract obstruction

**Figure 1:**
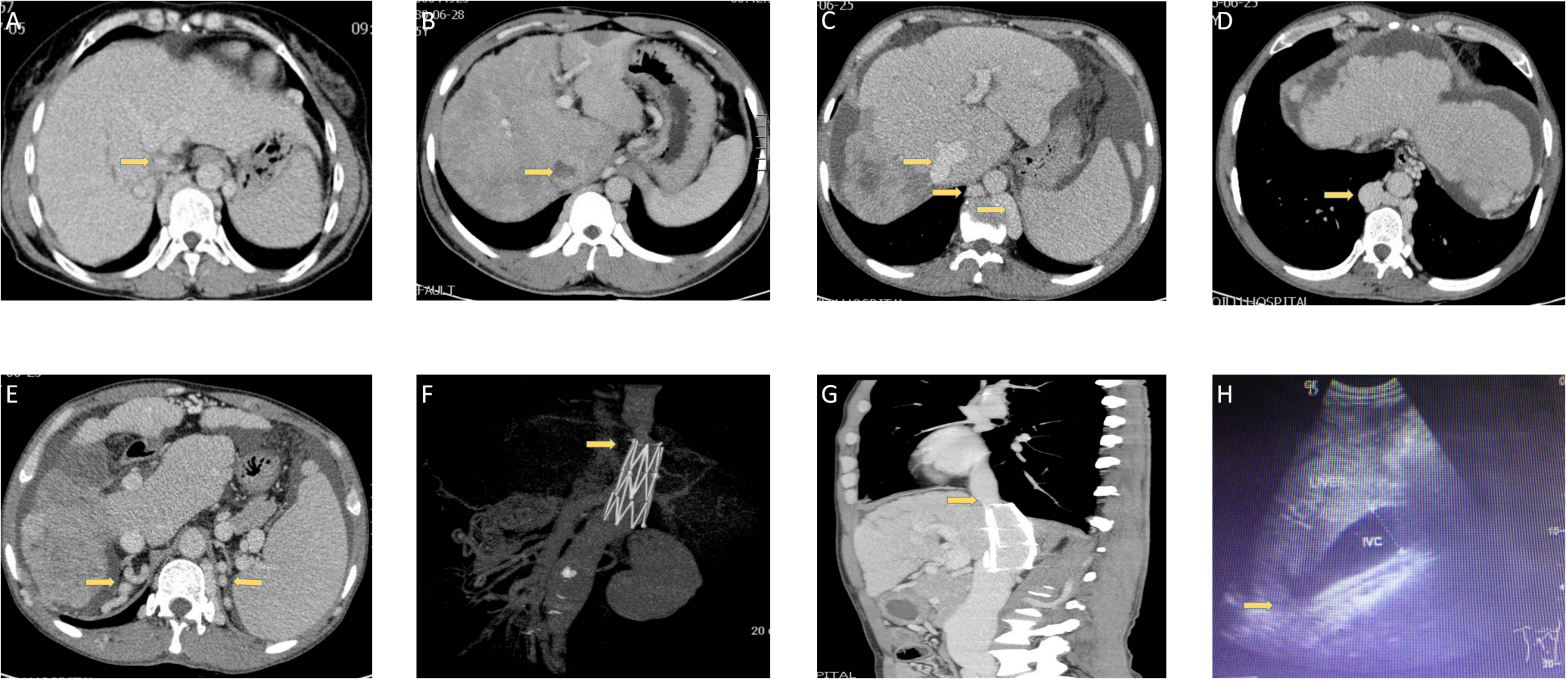
Different kinds of obstructions in BCS patients are complicated with HCC. **(A)** The narrowness of IVC in one patient. **(B)** Thrombosis in one patient. **(C, D)** IVC vascular malformation in one patient. **(E)** Compensatory vascular varicosity in one patient with no signs and symptoms. **(F, G)** CT angiography and a coronal section showing the segmental obstruction of IVC and corrected by stenting in one patient. **(H)** Ultrasonic imaging in one patient.

Different kinds of obstructions in BCS patients complicated with HCC are summarized in **Figure 1**. Segmental obstruction of IVC (**Figure 1A**), IVC thrombosis formation (**Figure 1B**), IVC vascular malformation (**Figures 1C** **, D**), as well as compensatory vascular varices with no signs and symptoms (**Figure 1E**) were observed in our series. **Figures 1F** **, G** were CT angiography and a coronal section showing the segmental obstruction of IVC and corrected by stenting, indicating a practical function of CT angiography in diagnosing BCS. IVC enlargement and narrowness of the hepatic part of IVC were also observed in our series. (**Figure 1H**)

### Imaging features of hepatocellular carcinoma

**Figure 2:**
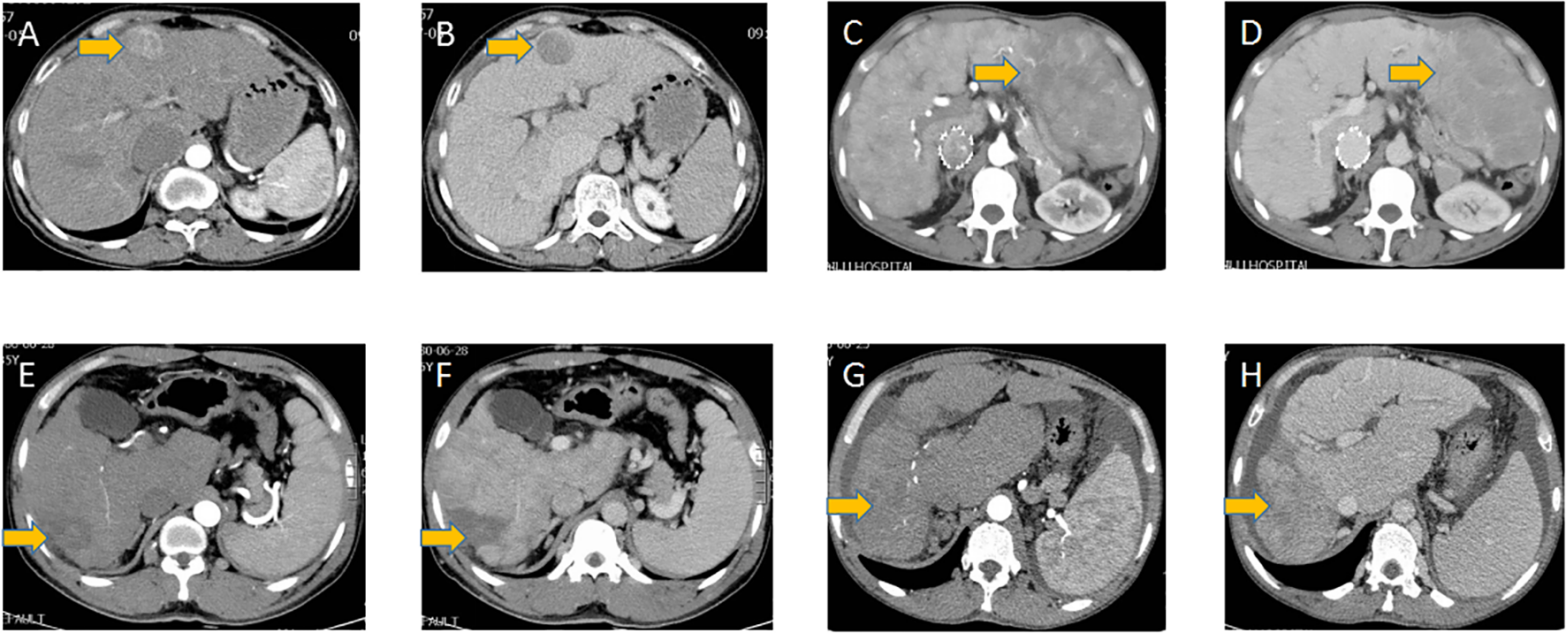
HCC tumors in different patients. **(A, C, E, G)** Arterial phase of HCC tumors in different patients, **(B, D, F, H)** the according delayed phase of HCC tumors in these patients.

The imaging features of hepatocellular carcinoma are shown in **Figure 2** and summarized in **Table 5**. Liver cirrhosis was observed in all patients. The tumor size was 0.9 cm, 7.5 cm, and 9.7 cm for the three patients with normal AFP levels, whereas the mean tumor size was 7.4 cm (range, 3.1-14 cm) in the other seven patients. 2 patients with tumor size over 10 cm were observed with intrahepatic metastasis. And one patient with the largest tumor size was observed with portal vein invasion, while no bile duct invasion was observed. The tumor nodules located in the left lobe of the liver of three patients and the right lobe of the liver of the remaining nine patients. And among these nine patients, five located in the right posterior lobe may indicate an association between the anatomical position and the development of HCC in BCS patients. All patients were single nodules, while 2 of 3 patients with normal AFP levels were multiple nodules.

**Table 5:** Tumor features of 12 HCC patients associated with BCS.

Pt#	LC	Tumor size (cm)	Tumor position	Nodules type	Growth pattern	PV invasion	BD invasion
1	+	3.1	Right posterior lobe	single	Expansion	–	–
2	+	3.1	center lobe	single	Expansion	–	–
3	+	0.9	Right posterior lobe	multiple	Expansion	–	–
4	+	7.5	Right posterior lobe	multiple	Expansion	–	–
5	+	9.6	Right posterior lobe	single	Expansion	–	–
6*	+	13.5	center lateral lobe	single	Expansion	–	–
7	+	5.3	Right posterior lobe	single	Expansion	–	–
8	+	NA	Right lobe	single	Infiltration	–	–
9	+	3.2	center medial lobe	single	Expansion	–	–
10	+	NA	Right lobe	single	Infiltration	–	–
11*	+	14	Right lobe	single	Expansion	+	–
12	+	9.7	Right lobe	single	Expansion	–	–

The largest nodule was selected to evaluate the nodule’s size, location, and shape if the patient had more than one nodule. *: with intrahepatic metastasis. LC: Liver cirrhosis; PV: portal vein; BD: bile duct.

The arterial and delayed phases of tumor nodules are shown in **Figure 2**. All nodules are located near the edge of the liver. All nodules exhibited irregular and heterogeneous enhancement in the arterial phase. Washout on the delayed phase occurred in all nodules.

### Treatment

The treatment of 12 patients with HCC is shown in **Table 6**. 2 patients underwent surgical portosystemic shunt, four underwent IVC stenting, one underwent surgical removal of membrane obstruction, and one underwent catheter-directed thrombolysis for hepatic vein and inferior vena cava block. There was hepatic resection for one patient, radiofrequency ablation for one patient, and TACE treatments for another five patients. The remaining patients refused related treatment and only received conservative treatment.

**Table 6:** Treatment of HCC and BCS in 12 patients with HCC.

Pt#	Treatment for BCS	Treatment for HCC
1	surgical portosystemic shunt+ IVC stenting	Hepatic resection
2	Conservative treatment	Hepatic resection
3	Catheter-directed thrombolysis+ angioplasty after RFA	RFA
4	Surgical removal of membrane obstruction+ IVC stenting	TACE
5	Conservative treatment	TACE
6	IVC stenting+ surgical portosystemic shunt	TACE
7	Conservative treatment	TACE
8	Conservative treatment	TACE
9	Conservative treatment	Conservative treatment
10	Conservative treatment	Conservative treatment
11	IVC stenting	Conservative treatment
12	Conservative treatment	Conservative treatment

## Discussion

HCC has been regarded as one of the significant complications of BCS. (5, 8, 11, 14–19) The exact pathogenesis of HCC in patients with BCS has not been elucidated. (20) It has been reported that the female gender was a risk factor. However, in our cases, the male gender is prominent. This may be due to the small sample quantity and sporadic cases. In accordance with previous reports, hepatitis viruses have also shown less association with the development of HCC in BCS patients. (15) Obstruction of the hepatic venous outflow tract leads to sinusoidal congestion, ischemic injury to liver cells, and portal vein hypertension, subsequently leading to hepatic congestion with necrosis, regeneration, fibrosis, and liver cirrhosis was believed to be one of the mechanisms. (14) Many other researchers reported and analyzed the imaging features of hepatic venous outflow tract obstruction. (21) However, the Chinese BCS population differed significantly from the western BCS population. (22) In our cases, all cases were with IVC obstruction, which is the same as the previous report. However, 4 of our cases developed HCC after curative IVC stenting treatment patients, and the obstruction forms in our series vary from case to case. This may indicate a completely different underlying mechanism. Hana Park et al. previously reported the association between hepatic vein pressure gradient and the development of HCC. (23) Thus, cases after IVC stenting may remain a high hepatic vein pressure gradient. And if this is the case, surveillance of the hepatic vein pressure gradient should be performed.

As for tumor characteristics, there is evidence of different mechanisms from the influence of hepatitis viruses, alcoholism, autoimmune diseases, or chemical intoxication. All the tumor nodules are located at the edge of the liver-75% of nodules located in the right lobe, especially the right posterior lobe. The pathology of those two patients after hepatic resection shows high differentiation, and in accordance, the invasiveness of the tumor seems only associated with the diameter of the nodules. Only in 2 patients with a diameter larger than 10 cm were the intrahepatic metastatic observed, and only the largest was with PV invasion.

The prognosis of HCC is still poor. Hepatic resection remains an effective and optimal choice for treating BCS-associated HCC in patients with A or B Child-Pugh grades. However, TACE and RFA have also been reported as effective treatment options. (11, 15, 18, 24, 25) Immune checkpoint inhibitor has been recently successfully applied in HCC patients. However, current knowledge about the application of immune checkpoint inhibitors was mainly obtained in HBV or HCV-associated HCC patients. Further studies should be performed to clarify whether BCS-associated HCC patients could benefit from immunotherapy. The treatment selection may differ in different cases, and the treatment strategy still needs further clarification.

## Data availability statement

The original contributions presented in the study are included in the article/supplementary material. Further inquiries can be directed to the corresponding authors.

## Ethics statement

The studies involving human participants were reviewed and approved by the Ethics Committee of Shandong University Qilu Hospital. The patients/participants provided their written informed consent to participate in this study.

## Author contributions

K-sL collected and analyzed the data. SG and K-sL analyzed the references and wrote the paper. Y-xC read the paper and provided revising advice. Z-lZ contributed to study supervision and revised the manuscript. All authors read and approved the final manuscript.
